# Editorial: New Insights Into Thymic Functions During Stress, Aging, and in Disease Settings

**DOI:** 10.3389/fimmu.2020.591936

**Published:** 2020-10-22

**Authors:** Nicolai S. C. van Oers, Dong-Ming Su, Ann P. Chidgey, Jarrod Dudakov

**Affiliations:** ^1^Department of Immunology, The University of Texas Southwestern Medical Center, Dallas, TX, United States; ^2^Department of Pediatrics, The University of Texas Southwestern Medical Center, Dallas, TX, United States; ^3^Department of Microbiology, The University of Texas Southwestern Medical Center, Dallas, TX, United States; ^4^Microbiology, Immunology and Genetics, University of North Texas Health Science Center, Fort Worth, TX, United States; ^5^Department of Anatomy and Developmental Biology, Monash University, Melbourne, VIC, Australia; ^6^Fred Hutchinson Cancer Research Center and the University of Washington, Seattle, WA, United States

**Keywords:** thymus, thymic tissue regeneration, thymic involution, severe combined immunodeficiency, tissue regeneration, FoxN1 gene, thymic epithelial cell (TEC)

The thymus is a bi-lobed lymphoid organ localized above the heart whose primary function is to foster the development of the T cells of the adaptive immune system. T cells are a critical component of the cellular immune system, helping B cells produce antibodies, releasing cytokines to orchestrate effective immune responses, and killing infected cells and/or neoplasm/tumor cells. The recognition of an infected cell or tumor cell, and the ability to support B cell secretion of antibodies requires T cells express a cell surface receptor, termed the T cell receptor (TCR). This receptor selectively binds to peptides complexed to major histocompatibility complex antigens (MHC class I and II). MHC class I molecules are present on all nucleated cells in the body, while MHC class II is restricted to antigen presenting cells. In the thymus, thymocytes rearrange DNA loci comprising the genes encoding the TCR subunits. Using a process termed VDJ recombination, each thymocyte expresses a unique TCR with exclusive recognition specificities. However, only those thymocytes expressing TCRs that can engage self-peptides/self MHC complexes present on thymic epithelial cells (TECs) are “selected” to form the peripheral T cell repertoire. This involves processes coined positive and negative selection, with the ensuing repertoire of T cells capable of recognizing pathogen- or distinct tumor- derived peptides presented on self-MHC molecules without overt reactivity to self-peptides. The critical role of the thymus in this process is best exemplified with infants born with mutations in genes required for the formation of the thymus (22q11.2 deletion syndrome or DiGeorge). No thymus results in no T cells, leading to a severe combined immunodeficiency. A second excellent example pertains to the spontaneously arising nude mouse, which lacks a thymus and hair due to mutations in the *Forkhead Box N1* transcription factor. This transcription factor supports the development of TECs, the controllers of T cell fate, and defective TECs means no T cells.

Yet, as late as 1960, the thymus was a scientific conundrum. Dramatic variations in its size in humans and diverse species had been documented for over two millennia. Severe clinical repercussions based on these size differences ensued due to a complete lack of knowledge about its functions, which was not revealed until demonstrated in pivotal experiments performed by Jacques Miller using thymectomy and skin grafting. These experiments established the thymus as the site of T cell development. For this, Miller was awarded the Lasker Prize in Biomedicine in 2019 (1). Between the 1890s–1950s, an enlarged thymic tissue noted in newborns and infants was proposed causal to asthma and crib death (thymicolymphaticus) (2). This unsubstantiated pathological connection led to tens of thousands of infants receiving “therapeutic” radiation treatments of 0.2–2 Gy to wipe out the thymus (3). Such an ill-conceived therapy resulted in >10,000 children developing thyroid cancers. A subsequent and less damaging treatment to reduce thymus size involved steroid injections. While not as severely marred as with the high radiation doses, the thymus could again be rendered hypoplastic. These therapies have revealed the extreme stress sensitivity of the thymus, but also its remarkable capacity for repair. In a normal setting, thymic function reaches its natural peak during the neonatal and pre-adolescent period, thereafter the thymus begins to decrease in size and activity. This process, termed thymic involution, results in significant loss in the production of T cells although some T cell development continues throughout adult life. This dynamic process, both with chronic age-related declines and acute atrophy caused by steroids or cytoreductive chemotherapy, can have profound negative impacts in the efficacy of adaptive immunity. Today, understanding how to maintain the function of the thymus through-out life, particularly after damage, is the driving goal of researchers.

In the series of articles under the title “New Insights into thymic functions during stress, aging, and in disease settings” in this issue of Frontiers in Immunology, novel insights and findings by leading scientists in the field of thymus biology are presented. The articles include two reviews outlining recent discoveries into the mechanisms required for the formation and specification of the thymus within the 3rd pharyngeal pouch during embryogenesis (Bhalla et al.; Giardino et al.). The contributions of key transcription factors such as *T Box Transcription Factor 1* (*TBX1*) and *FOXN1* in the patterning of the pharyngeal apparatus and the subsequent demarcation of the thymus anlage are discussed in conjunction with the differentiation and expansion of thymic epithelial cells. The formation of the two different populations of epithelial cells, cortical and medullary TECs and their selective roles in supporting and educating thymocytes to form the repertoire of T cells, which is unique to each individual, is described, revealing new subsets of these cells in the process (Alawam et al.).

As is well-recognized currently, in addition to aging, the thymus undergoes a transient and sometimes massive reduction in overall size and cellularity following infections, corticosteroid production through the hypothalamus-pituitary-adrenal axis or via injections, and chemotherapy treatments. The impact on thymocytes and stromal cell populations are presented herein (Gaballa et al.; Kinsella and Dudakov). Such thymic hypoplasia is widespread in the population, and the different conditions leading to hypoplasia/aplasia of the thymus are presented in a combination of research and review articles in this series (Cowan et al.; Gaballa et al.; Pérez et al.; Silva-Freitas et al.) For example, thymic hypoplasia is evident in pregnant women and infants born prematurely. The natural aging process causes a gradual and “permanent” hypoplasia of the thymus and this involves both loss of developing T cells and TEC subsets, with a greater impact evident in males than females, at least until middle-age (Hun et al.). The epithelial tissue within the thymus undergoes adipogenesis and epithelial to mesenchymal transitions (Lavaert et al.). Given these varied and damaging processes, improving and/or restoring thymopoiesis, from infants to the elderly, requires new therapeutic strategies. Several innovative approaches covered in this series include use of pro-T cells to improve T cell formation in the thymus (Singh et al.) and the use of specific cytokines to restore TEC functions (Kinsella and Dudakov). Humanized mouse models and comparative mouse-human studies offer additional understanding and methodologies for improving thymus functions (Machado et al.; Tong et al.).

The emergence of checkpoint blockade immunotherapy to treat cancer patients has consequences as well, since the incidence of autoimmune complications with such a patient cohort increases dramatically due to the antibody treatments (Wang et al.). While the thymus is usually considered in the context of T cell development, there is evidence of some B cell development in normal and clinical settings. Two pronounced conditions, the autoimmune diseases, Systemic Lupus Erythematosus and Myasthenia Gravis, have substantial levels of B cell development/expansion (Hidalgo et al.). In the case of Myasthenia Gravis, thymectomy is actually done for patients to eliminate the autoreactive B cells in the thymus, improving their clinical outcomes. Alzheimer's Disease is another clinical condition where mouse models have established that intrathymic injections of TECs, derived from stem cells, can actually improve cognitive functions (Zhao et al.). In summary, we have come a long way from purposely destroying the thymus to focusing on its restoration and regeneration. These are nicely collated in our Frontiers in Immunology Topic.

## Author Contributions

All authors participated in the editorial reviews of the manuscripts. All authors participated in the writing and editing of the editorial article.

## Conflict of Interest

The authors declare that the research was conducted in the absence of any commercial or financial relationships that could be construed as a potential conflict of interest.

## References

[1] Miller JF. A scientific odyssey: uncovering the secrets of thymus function. Cell. (2019) 179:21–6. 10.1016/j.cell.2019.08.023 31519307

[2] Dally A. Status lymphaticus: sudden death in children from “visitation of God” to cot death. Med Hist. (1997) 41:70–85. 10.1017/s0025727300062049 9060205 PMC1043871

[3] Jacobs MT Frush DP Donnelly LF. The right place at the wrong time: historical perspective of the relation of the thymus gland and pediatric radiology. Radiology. (1999) 210:11–6. 10.1148/radiology.210.1.r99ja4511 9885579

